# From Bystanders to Upstanders: Supporters and Key Informants for Victims of Gender Violence

**DOI:** 10.3390/ijerph19148521

**Published:** 2022-07-12

**Authors:** Lidia Puigvert, Marta Soler-Gallart, Ana Vidu

**Affiliations:** 1 Department of Sociology, University of Barcelona, 08034 Barcelona, Spain; lidia.puigvert@ub.edu (L.P.); marta.soler@ub.edu (M.S.-G.); 2 Department of Sociology, University of California, Berkeley, CA 94720, USA; 3 Department of Private Law, University of Deusto, 48007 Bilbao, Spain

**Keywords:** bystanders, upstanders, social impact, gender violence, isolating gender violence

## Abstract

Scientific literature has presented relevant evidence about the existence of gender violence in science and has evaluated some programs and actions against this problem. Although many researchers have identified the importance of those intervention programs to overcome this harassment, it is still a predominant reality in institutions, surrounded by the law of silence. Emerging lines of research are studying which of those programs are successful in this endeavor, and their transferability to other contexts. This research has analyzed one program: Programme of Women’s Dialogic Action (ProWomenDialogue). To gather evidence for expressing whether or not ProWomenDialogue has an impact, and whether it constitutes a successful action against harassment, the SIOR (Social Impact Open Repository) criteria, emerging from the FP7 IMPACT-Project, have been used for the evaluation of this research’s social impact. Drawing on SIOR, ProWomenDialogue shows unprecedented transformations in academia through six lines of action. The political impact led to legislation that made compulsory the creation of equality committees and protocols against sexual harassment. Social impact, aligned with SDG 5, inspires the reduction of GBV, while encouraging the career promotion of female researchers. ProWomenDialogue embodies a Successful Action platform against violence, presenting their features as recommendations to be implemented in other settings.

## 1. Introduction

Best research has already shown that scientific excellence cannot be separated from human excellence. However, building an academic environment free from gender violence and committed to scientific and ethical values is still a challenge in many universities around the world. Sexual harassment and gender-based violence (GBV) constitute a concerning reality worldwide. Its incidence is alarming, not only because of the high data but also because of the different areas and contexts in which it occurs. According to the report of the World Health Organization [1], 1 in 3 women worldwide (35%) have suffered some type of physical or sexual violence throughout their lives. There is no institution or sector immune to harassment. It occurs in companies, universities, factories, the film industries, congresses, and in society in general. Universities, similar to any hierarchical institutions, may also have cases of sexual harassment [2]. This is confirmed by data: 1 in 5 women are sexually assaulted in the university [3,4]. Gender equality will not exist as long as sexual misconduct continues. Power relations, institutional hierarchical structure, and a mostly adverse environments for women in the workplace make possible the existence of such behavior [5]. In this article, we will use sexual harassment predominantly as a term to refer to sexualized practices that are unwelcome, but it may also encompass a range of other unwelcome conduct, such as the use of sex-based language and imagery at the workplace. Sometimes, it may involve sexual assault practices that may occur in a workplace context. The terms, gender violence, gender-based violence, and violence against women, are usually used to refer to physical and psychological abuse more generally. When one of these broad terms, such as gender violence, might include sexualized practices, the rubric “sexual violence” is used to include this broad range of meanings.

The first study that analyzed gender violence in Spanish universities concluded that 62% of university students knew of or had experienced sexual harassment situations at their colleges [6]. As international research has demonstrated [7], the creation of a favorable context for reporting harassment is essential to ensure that victims feel supported in filing a complaint against undesirable conduct [8]. Hence, the need for peer support, and especially bystander intervention is one of the most effective responses [8]. Nevertheless, the power relations that underlie academic institutions have for a long time been understood as a barrier to reporting. Even more, for many victims in academia, the consequences of daring to break the silence have proved to have a worse impact on their health, well-being, and academic promotion than having themselves suffered sexual harassment [9,10]. Those female and male researchers, and academics who have supported the victims in their struggle to denounce, due to “feudal” power relations, have also suffered what is known as ‘Isolating Gender Violence’ (IGV) [11], aimed at preventing them from bringing to light the facts hidden until then. Back in 1990, Dziech and Weiner [12] analyzed the reasons why the lack of complaints was still so apparent. SOSH came up as another important element when it comes to examining why complaints hardly occur. Actions such as criticisms, reprisals, and attacks that those who support survivors may receive, restrict the number of complaints, as victims often feel that they would rather not have support. This reality embodies a social concern, since it is thanks to such support that survivors may come forward.

Many scholars have analyzed well the impact of the “glass ceiling” (unofficially known as a barrier to advancement in a profession, mostly affecting women) on female researchers and scientists, showing the negative consequences on their career promotion [13,14,15]. However, it remains poorly studied, at both the international and the Spanish levels, how sexual harassment in academia creates a hostile context, especially (but not only) for female researchers and scientists. It supposes a burden on their promotion, leading them in extreme cases to quit what would have been excellent scientific careers, or to make decisions that can also have a negative impact on their careers (e.g., change from prestigious departments and not accepting positions of power, among others). Drawing on this, major advancements have been made in Spanish universities since early 2000, and even before, in breaking the silence about sexual harassment, thus shifting the balance of power from hiding and rejecting the very existence of cases to recognizing that, as expected, they also occur. This makes it necessary to create complaint mechanisms as well as support mechanisms for victims. The struggle against sexual harassment in Spanish universities has also been possible due to the organization of students and female academics hand in hand with egalitarian men who dared to challenge institutional resistance [16]. Indeed, research projects such as the UniswithHeart project [17] are building on this to continue working on creating networks of support, providing evidence to encourage bystanders to become upstanders and inspiring victims to become successful survivors.

The Community of Researchers on Excellence for All [CREA] [18], a network of researchers that currently gathers together around 70 researchers and academics from different disciplines (sociology, education, economy, history, anthropology, neuroscience, psychology, among others) from different Spanish universities, and especially its Women’s Group, SAPPHO, has had a crucial role in this struggle, developing, since 2003, a Programme of Women’s Dialogic Action (hereinafter, ProWomenDialogue), aimed at breaking the silence and transforming feudal university structures that promote sexual harassment, contributing to victim’s support and well-being, as well as acting for those students of universities that adolescent girls, and their families, dream of for their future.

In this article, we present the three characteristics of this programme and discuss the social and political impact it has achieved, following the six lines of action it embodies. This sheds light on how the ProWomenDialogue Programme has contributed over the years to break the silence about sexual harassment in universities and institutions.

### 1.1. Retaliations for Complaining

While the numbers of GBV cases are high, the data on what it is still unknown is also very high, as one of the barriers identified in the literature about cases that are followed, are the reprisals suffered by both victims and victims supporters in this process [19]. Retaliatory attacks and other negative consequences are directly associated with reporting as several studies have already shown [20]. Specific examples of attacks and negative repercussions on the academic career of victims have been broadly published in scientific articles [2,21]. Policies and actions developed by Spanish universities need to be grounded on the principle of zero tolerance toward any kind of violence against women, as well as on the principle of support and solidarity with survivors and their supporters [12]. Even though policies and committees of equality have been created within institutions and some structural changes were achieved, the overcoming of sexual harassment in academia is still a challenge. Because of pioneer research, universities were pushed by law to create equality protocols [6]. Indeed, an issue to be accomplished states people’s commitment and action, i.e., whose who in charge of these protocols, since they are not always standing up for survivors. Scientific research has also shown resistance suffered by feminists and academia when struggling to free universities of sexual harassment [5]. This reality has indeed led to the identification of the isolating gender violence directed at those people who have placed themselves against academic harassment and in favor of survivors, experiencing negative consequences for speaking out and supporting direct victims.

### 1.2. Successful Actions to Tackle Sexual Harassment: Bystander Intervention

Some of the most successful actions in the university environment are in line with supporting survivors. The direct or indirect support and intervention of all those who see or have knowledge of a case are what makes a person feel supported. Most research in the analysis of the mechanisms that have been most successful in preventing and responding to GBV in universities and organizations has to do with the bystander’s role [8]. Slogans such as “be an active bystander”, “take the pledge”, “if not you, who?”, “if, not now, when”, “see something, say something”, raise awareness of the so-called historical social moment to be on the victim’ side [17]. Bystander training involves empowering victims to stand up, encouraging colleagues, friends, professors, administration, and other bystanders to report harassment incidents rather than overlook them, thus, creating an academic environment that prevents and responds to any kind of undesired behavior and sexual harassment. In this vein, in this article, we seek successful actions that has been shown to have a good impact once previously applied. By Successful Actions, we understand such actions that lead to achieving impact [22,23], which shows the best results (not only good results) in a variety of contexts and countries. The bystander intervention program [20] constitutes an example of successful action, because over several years, in several places and a variety of contexts, it proved to be the best way to tackle sexual harassment in schools, universities, and institutions. However, it is not enough to be a bystander. To help and support survivors, intervention is needed. Nevertheless, according to this study, 40% do not report because of fear of retaliation [24]. Therefore, it is important to create a culture of response by responsible bystanders in which upstanders are protected against what is known as IGV. The COVID-19 context, which exposed the bystanders’ role as crucial also in the virtual sphere, has also accelerated the urgency to research mechanisms that encourage and protect upstanders.

### 1.3. Networks of Solidarity

Solidarity networks constitute a crucial element in empowering survivors, protecting those who support victims of GBV and so challenging universities by fighting against sexual harassment. The Solidarity Network of Victims of Gender Violence at Universities [25], currently called MeToo University [16], was the first peer-to-peer solidary network in Spain, created in 2013 by survivors and activists, to provide support for direct victims of sexual harassment and for those who support them. This Solidarity Network was a turning point in the way universities and other people faced harassment, identifying it as a problem and deciding to act against it. At some point, the distinction between behaviors that constitute or do not constitute harassment is unclear. This confusion, together with a hostile atmosphere may lead to creating a feeling of blame and responsibility in survivors. Usually, it is associated with a lack of support and fear of reprisals and criticisms, such as being considered a troublesome person [10]. Victims face many difficulties in breaking the silence and moving forward [17]. Victims cannot cope on their own. Barriers such as self-doubt, external questioning of disclosure, social isolation or victim-blaming explain why approximately 88% of cases are still not reported [4]. If this percentage is high in a normal situation, and despite the many mechanisms already in place, in a pandemic situation, which led to confinement, many of these cases never became known. The pandemic highlighted an already evident problem. It raised awareness that many victims were confined with their abusers, women as well as children [26]. Survivors’ support in the pandemic context is especially important.

### 1.4. Women in Science. Women at the Workplace

Universities are institutions that embrace relations of power and privilege at the same time as knowledge and values. However, both spheres are related in order to be successful as an institution [6,17]. Harassment impairs the functioning of institutions and the people who form them. Conversely, harassment-free relationships guarantee the integral development of companies and their workers and, therefore, of society as a whole [13,14]. In recent decades, the treatment of people and their active roles in institutions have changed enormously [24]. A potential atmosphere of harassment at the workplace may imply negative consequences not only for employees but also for the organization of work and the achievement of business productivity goals [27]. Human excellence adopted a new direction in what we currently call business culture. The environment and our perception of it now play a key role in human behavior and, also, in how people act in their daily lives [15]. In the same line, it has been shown that the way in which a company deals with equality issues determines not only a good working environment and the well-being of its workers, but also its benefits in the market [28,29].

### 1.5. GBV in the COVID-19 Pandemic

The COVID-19 pandemic has unleashed a global health concern with devastating consequences for the population, especially for the most vulnerable groups [29]. Beyond the global health problem, 6.28 M people died up until 27 May 2022 [30], the reality of gender-based violence has caused a problem and aggravated this reality in different contexts. According to UN nations, GBV has increased during the pandemic due to factors such as health, security, and money worries, isolation from the abusers, cramped living conditions, movement restrictions, and deserted public spaces [31]. Facing this reality, several mechanisms concerning GBV and survivors’ protection started to be reviewed and reinforced during the lockdown. The Hackathon EUvsVirus constitutes one example in this regard [28], wherein the EC asked researchers from different countries and disciplines to join forces and build together solutions to support potential victims of GV during the lockdown. In the same line, the “open doors” initiative was built as a mechanism to prevent child abuse during the confinement caused by the pandemic [26]. The Roma women students’ gathering was held online as an alternative to face-to-face meetings and continued conducting activities during the pandemic using their strong sense of resilience to overcome challenging situations [32]. This article purports to show how supporters and key informants are relevant and, sometimes, the unique agents are available to help survivors, which becomes crucial in extreme contexts, such as the one created by the pandemic and all that breaking the silence on GBV may involve in that setting. Thus, the COVID-19 context becomes an essential scenario, not only with the requirement for trustworthy and helpful health information [33], but also to gather knowledge on how someone may cross that line from bystander to upstander.

## 2. Materials and Methods

### 2.1. Presenting the Programme of Women’s Dialogic Action: Breaking the Silence about Sexual Harassment in Spanish Academia

In 2003, the CREA-SAPPHO research group joined the ‘Unitary Platform against Gender Violence’ formed by 121 entities [34]. Being at the time the only research group member of the Unitary Platform, the Programme of Women’s Dialogic Action (ProWomenDialogue) was one of its contributions, aimed at breaking the silence and transforming feudal structures, and universities, which permitted sexual harassment to happen [5]. It is important to underline the fact that this article only depends on the CREA-SAPPHO approach in the university context, as it covers many other areas of GBV prevention and response, and the research group was created long before its inclusion as a member of the “Unitary Platform”. In fact, as developed below, the ProWomenDialogue designed six main lines of action, all of which have been developed for decades, as this Program is part of a line of research and action that started back in the nineties:

1. First, the development of a scientific line of research on GBV in universities, oriented not only to research about existing sexual harassment in academia, but also to actions and strategies that contribute to its overcoming.
2. Second, actions aimed at influencing the policy level, thus opening up the debate on sexual harassment in universities at the Parliament and beyond, achieving political representatives (from several regions and different Autonomous Communities in Spain, underlining Madrid and Catalonia) to legislate on this issue.
3. Third, actions oriented to impact university policies focused on changing feudal structures and, therefore, removing the institutional support that harassers have historically had in Spanish academic circles.
4. Forth, reporting existing cases of sexual harassment.
5. Fifth, actions focused on supporting victims in two ways: ensuring that those who wanted to file a complaint could do it while looking for ways to protect them from the negative consequences that this action may involve for their personal lives, their physical and mental health, as well as on their academic and scientific careers.
6. Sixth, the development of a scientific line of research on isolating gender violence (potential criticism, attacks, and harassment that those who support victims my suffer just because of doing it), and the reporting of existing cases.

With the endeavor of developing the above-mentioned set of actions, ProWomenDialogue was built upon three main characteristic features.

1. The first characteristic of ProWomenDialogue consisted of opening up the debate about sexual harassment in academia to all sectors of society, including political representatives from different political parties. This way, representatives of different social, institutional, and political spheres were invited to participate in multiple actions organized under the framework of the programme. This fact set the context to speak up about poor career progression and low representation of senior women leaders in science [35], and its connection with sexual harassment in the Spanish academic context, as well as the needed institutional policies to change the situation.
2. The second characteristic emphasized dialogue with all women, including those with no academic credentials, and women from the largest ethnic minority in Spain who have been traditionally excluded from academic spaces, such as Romani women [36]. ProWomenDialogue set up spaces to dialogue not only with female academics and researchers, but also brought into these spaces the voices of the ‘Other women’ [37], who, due to not having university degrees, have been traditionally left at the margins of traditional feminist debates. This term is what dialogic feminism refers to [38]. The ‘Other women’ movement has been essential in the struggle against sexual harassment in universities as, differently from other existing movements within universities, they have neither feared reprisals, nor been subdued by those who had power positions and were harassers, abusing their power and privilege. Besides, many of these women have daughters and granddaughters enrolled in colleges, which has been a major force for them to support the actions of the programme.
3. The third characteristic of ProWomenDialogue was to find allies in the struggle against gender-based violence and sexual harassment in universities with egalitarian men, hence, dialoguing with them about the actions and strategies that needed to be embraced. Historically, women and men have led feminist conquests, jointly, while they have also encountered barriers posed by some women and men who want to maintain the status quo. The struggle for violence-free academic spaces also needed the joint action of feminist women and egalitarian men. CREA-SAPPHO researchers collaborated with egalitarian and diverse men, including representatives of Roma associations or sexual minorities such as LGBTIQ+, which also contributed to shedding light on the discrimination they suffer on college campuses [39]. Within CREA, the dialogue with men was materialized in the collaboration with the association Men in Dialogue, which works for the promotion of alternative models of masculinities that break with traditional dominant masculinities [40].

### 2.2. Selected Tool

This study presents and discusses evidence of one of the research tasks carried out in the framework of the IMPACT-EV Project (FP7 from the European Commission) [22] that in charge of creating a system for the evaluation of the different impacts of social sciences research, thus, making possible the identification of Successful Actions in research leading to social impact (those actions that scientific evidence has demonstrated obtain success in different social contexts, and are transferable, in relation to the objectives democratically decided by citizens) [23]. One of the outcomes of IMPACT-EV was the creation of the first Social Impact Open Repository (SIOR), a tool that facilitates and promotes researchers to show and share the social impact of their projects with other researchers or stakeholders [41]. Drawing on SIOR, qualitative evidence of the political and social impact of ProWomenDialogue has been collected and analyzed. Based on the previous definition of the social impact of research [42], SIOR established the following criteria for the evaluation of political and social impact:

- connection to United Nations Sustainable Development Goals, EU2020 targets, or other similar official social targets;
- percentage of improvement achieved in relation to the starting situation;
- replicability of the impact: the actions based on the project findings have been successfully implemented in more than one context;
- publication by scientific journals (with a recognized impact) or by governmental or non-governmental official bodies;
- sustainability: the impact achieved by the action based on project findings has shown to be sustainable throughout time.

### 2.3. Data Collection

The task consisted of collecting and analyzing qualitative evidence of both the political and social impact of ProWomenDialogue. It is important to highlight that, for this article, we only analyze the ProWomenDialogue model in relation to higher education and the struggle against sexual harassment in this specific context. Tracking the impacts of the study made it possible to unveil the achievements of the programme in the struggle against gender-based violence and sexual harassment in Spanish academia between 2003 and 2022. The data collection strategy used is based on Documentary Analysis. Evidence considered for this study was gathered from a wide set of sources from published scientific articles in ISI Web of Science and SCOPUS database, existing legislation, media news published in general-interest newspapers (Spanish and international press), working papers, and reports of scientific research about GBV in Spanish Universities, as well as doctoral dissertations about sexual harassment in universities. The period, for which evidence has been gathered, corresponds to the lifespan of the Programme of Women’s Dialogic Action, the experience analyzed in the study, between 2003 and 2022.

### 2.4. Data Analysis

Analysis of the political and social impact of research was conducted using SIOR as an instrument. In its conceptualization of social impact, the IMPACT-EV project distinguished between dissemination of research, transference of research, and social impact of research, setting up the basis to establish criteria, hence better identify when scientific research truly contributed with its knowledge to improving the social reality [43]. In this sense, beyond dissemination and knowledge transfer, the social impact of research is defined as the fact that when the published and disseminated research results, which have been transferred, lead to an improvement in relation to the goals agreed upon in our societies through our political representatives (for instance, the UN Sustainable Goals, EU2020 targets, or others).

For the qualitative data analysis, a system of categories emerged from the data, based on the following elements: political impact in society, political impact within academic institutions, and social impact. The process of data analysis that was conducted followed the communicative methodology of research [23], which involves analyzing the data according to, on the one hand, an exclusionary dimension (barriers that prevent actions from having successful results and social impact) and, on the other hand, a transformative dimension (positive elements that enable social impact). In the case of this article, we only focused on those transformative elements that help to identify successful actions that prevent and respond to sexual harassment in academia, and so can be transferred to promote social impact in other contexts.

## 3. Results

This study brings into the international sphere a ground-breaking programme that, due to its three characteristics (to dialogue with all sectors of society, including their political representatives; to dialogue with all women, including women with no academic credentials, and; in collaboration with egalitarian men, to dialogue with men) has broken the silence about GBV and sexual harassment in Spanish academia. In this way, ProWomenDialogue has generated a political and social impact that is changing the feudal hierarchies in Spanish universities. Reviewing existing evidence related to the actions developed in the framework of the ProWomenDialogue following the SIOR criteria has achieved an unprecedented transformation in both Spanish universities and science, breaking the silence on sexual harassment, especially relevant for empowering survivors and making them become successful survivors.

### 3.1. Political Impact on Society

As this study shows, a change in power relations has started in Spanish universities. This change has been promoted by the results of the first research project [6], changes in legislation, institutional improvements, and preventive strategies. As a milestone of ProWomenDialogue in the development of the scientific line of research on GBV, between 2005–2008, CREA-SAPPHO led the RTD national funded research study ‘Gender-based violence in universities’ [6]. This was the first study that investigated the topic of GBV in Spanish academia, daring to reveal quantitative and qualitative data on the issue. The ongoing dialogues that were triggered after 2003, when CREA-SAPPHO started to be part of the Unitary Platform Against GBV (it is important to highlight that the group had been working on this line for many years before this moment), and progressively as more actions have been conducted in order to better understand how the power-based academic relations worked, and had an impact on the political realm. In 2004, the Organic Law on Integral Protection Measures Against Gender Violence was passed [44]. However, despite the progressive character of this law, no mention of some key issues was comprised in the law. For example, the legal text did not mention two facts: the existence of GBV in sporadic relationships, and the universities as spaces in which violence could actually happen. Following a participatory process created by the Government of Catalonia, the Unitary Platform against gender violence contributed to the Law 5/2008, of 24 April, concerning the right of women to eradicate sexist violence. CREA-SAPPHO had been working for years on the prevention of violence in adolescence and violence in sporadic relationships, that is, gender-based violence existing in sexual-affective relationships outside the standard of a stable partner or ex-partner, and presented results in very diverse forms to academic, and also non-academic, spaces. Both of these issues were also contributions from our research to the Platform. CREA, through scientific evidence we had, proposed to the Platform that the term gender-based violence be considered also in the case of sporadic relationships, and to be included as such in the Catalan law [45]. The proposal was accepted in the assembly and the Unitary Platform took this proposal, among others, to the Parliament of Catalonia. In this way, GBV in sexual-affective relationships considered outside the couple was included in the law. Regarding GBV in universities, as discussed, CREA-SAPPHO colleagues reacted to this by launching a set of conversations with members of the Parliament from all political parties in order to present evidence to show how sexual harassment also occurred in academia, proposing to legislate the creation of Committees of Equality in all universities [46]. Those members of the Parliament made a public commitment to do it, which was reflected in the Organic Law 4/2007 through which the Organic Law 6/2001 of Universities was modified. The Organic Law 4/2007, of 12 April, introduced an additional provision regarding ‘Units of Equality’: Universities will count among their structures Units of Equality for the development of the functions related to the principle of equality between women and men [47].

Recently, the political impact has shown its relevant by highlighting the legislation that deals with the protection of those who protect, the IGV concept, in two autonomous communities in Spain. Catalonia was the pioneer to include into the Modification of the Catalan law 5/2008 of 24 April, the rights of women to eradicate sexist violence. Article 5.4 [48] states the definition of IGV as such: point (g) Second-order violence: consists of physical or psychological violence, retaliation, humiliation, and persecution against people who support victims of sexist violence. It includes acts that prevent the prevention, detection, care, and recovery of women in situations of sexist violence. Recently, the Basque Country Law 1/2022, of 3 March, acted on the second modification of the law for the equality of women and men [49], and also included IGV in the law under Article 50 defining it as follows: “4. Sexist violence against women is also considered violence against people who support the victims”. These legislations mark an important milestone in the fight against sexual harassment, empowering bystanders to become upstanders and making victim support an increasingly true reality. At the same time, this inspires other legislations, at the national and international levels, to do the same.

### 3.2. Political Impact within the Academic Institutions

Law 3/2007 indicates that companies with 50 or more workers must adopt measures to ensure equality, being these measures specified in an Equality Plan. The fact that universities are considered companies with more than 50 workers means these institutions are also forced to prepare an Equality Plan. One of the measures of these Equality Plans was, together with a set of evaluable measures aimed at removing the obstacles that prevent or hinder the effective equality of women and men, the prevention of sexual and gender-based harassment, as stated in Article 46 of the aforementioned Law 3/2007 [44]. The political impact achieved in society brought with it an unprecedented political impact within academic institutions as well. According to the evidence collected, legislation was passed regarding the compulsory requirement of creating, in all public universities, mechanisms to prevent sexual harassment. At the same time, the different public actions that were being deployed in the framework of ProWomenDialogue were oriented toward breaking the silence and set the ground for creating, for the first time in Spanish Universities’ history, Committees of Equality. Explicitly, one of its functions would be preventing sexual harassment and protecting survivors from all harmful consequences they may experience. Currently, most of the public Universities in Spain have Equality Committees and protocols against sexual harassment.

### 3.3. Social Impact: Breaking the Silence

According to evidence gathered, ProWomenDialogue actions set the context for students and researchers to dare to talk about existing gender-based violence and sexual harassment in Spanish academia. In this way, in 2011, a most talked about complaint was filed in Barcelona. The case was initiated by a junior female researcher who had been harassed on two occasions by the same catedrático: when she was a degree student (in 2008), and later on when she was a master’s degree student. The harasser was well-known as a Sociologist catedrático with a powerful position in the feudal Spanish university system, which had allowed him to be a public harasser during his entire career for more than 30 years [50]. In 2011, when the victim (junior researcher) decided to report the case, she asked for the support of her PhD advisor in filing the complaint. Her advisor was Dr. Ramon Flecha, the only full professor who, since 1995, had reported sexual harassment at the University of Barcelona (hereinafter UB) and, for his stand, he become a victim of what is known as Isolating Gender Violence. Flecha first filed the complaint (based on this young survivor’s proof), and then, 13 different students who had been victims in previous years joined and denounced the harasser professor. Flecha presented the complaint at the same time at the UB and at Harvard University because the harasser signed e-mails to his victims including the name of the latter institution as his signature. Initially, the UB decided that there ‘was no case’. After a while, the UB received an email from Harvard University and decided to re-open the case but so slowly that it ended up expiring. The prosecution resolved that evidence presented by the victims showed sexual harassment conducts, some of which are even of a reason to send him to prison. However, given the statute of limitation and the fact that the harassment had occurred more than three years before the complaint was handled by the prosecutor, the cases had to be closed [51]. After years of conflict, counting on international support, and the great job most journalists did, the situation finally changed at the university, and is now in favor of survivors.

Since then, the path has been open for other cases to be reported not only at the Committees of Equality, but also in Courts. Besides, following the precedent of North American universities and facing the lack of institutional action regarding the cases of sexual harassment at the University of Barcelona, the Solidarity Network of Victims of Gender Violence at Universities was created at the end of 2013. This is the first peer-to-peer initiative in Spain that emerged “from below”, by a platform of victims of sexual violence in Spanish academia (mainly the group of students reporting the first complaint at the UB previously mentioned) and by the people who support them, including victims of isolating gender violence. Since 2011, when the first complaint was filed, different cases of students, researchers, and academics have emerged and have been reported, many of them being spread in the press. Accordingly, in 2016 and 2017, cases were heard of in the University of Seville, the Complutense University of Madrid, the University of Barcelona, and many others became known.

### 3.4. Recommendations

Starting from the purposes developed by different campaigns carried out at some prestigious universities, as well as the results of research with the greatest impact on the subject, the following proposals for the prevention, attention, and eradication of sexist violence were obtained:

- Zero tolerance towards violence at the university. Involvement of the university as an institution and joint work of the entire university community to enhance the reporting of situations of sexual violence that occur within the university campus.
- Support and solidarity with survivors. Victims are never guilty. Facing possible guilty reactions and in unsupportive contexts, victims are less likely to explain the facts and seek help. Therefore, research concludes that it is necessary to create spaces for support, assistance, and solidarity within the university, to help survivors. At the same time, it is necessary to guarantee non-victimization principles and a lack of reprisals towards people who make situations of violence visible and ensure the protection of the person who has reported or filed a complaint.
- Protect IGV victims. Isolating gender violence means reprisals and repercussions that people who stand in solidarity and act in favor of victims might receive. These people may have different profiles, mediators, faculties, advisors, students, assistants, and generally, they are the people committed to reporting and helping direct victims.
- Guarantee professionalism in the treatment of violence. It must be ensured that any resource that has the purpose of addressing sexist violence works with scientific rigor, i.e., equality committees; action protocols; faculty training; sexual harassment prevention offices, and other actions deemed appropriate. Therefore, it is necessary: 1. to implement successful actions that have been carried out in other universities around the world in the field of prevention and intervention in cases of sexist violence within the university context; 2. people who are part of any of these resources have a proven record of accomplishment and commitment regarding prevention and intervention in situations of sexist violence. For instance, they may have an international trajectory in the study and prevention of sexist violence, and; a trajectory in complaints of sexual violence cases, breaking institutional silence, capacity for mobilization, and proposing ways of prevention and overcoming situations of violence. Finally, in order to guarantee transparency in selecting people in charge of assuring an ethical atmosphere at their workplace, their CVs and highlighted merits would be made public.
- To create confidential spaces, where victims or people who have witnessed a situation of harassment could explain it, while announcing the different actions that the university takes in these situations.
- To inform, in different ways and sites, physically and virtually, the services available at universities and to break the silence about this problem, creating awareness. In this sense, informative documents might be distributed to the entire university community, containing information on university services, advice, and examples of situations of sexual violence in order to be able to identify them easily. These materials may be shared, for instance, around the cafeteria, at parties, student dorms, library, and other spaces.

## 4. Discussion

Until a decade ago, in Spanish-dominant analyses about the causes that motivate female researchers to quit academia or not have the same opportunities as men, the predominant causes found have been those linked to motherhood or the unequal distribution of caring responsibilities [5]. Recognizing the huge cost that this supposes not only for women’s scientific careers but also for the entire society, a complete scientific analysis must also include other variables. In this article, we argue how sexual harassment may constitute an obstacle that leads female researchers to quit academia and worsen their physical and physiological conditions. Excluding this very issue from the analysis is grounded on the profound resistance to address and challenge the feudal dynamics in which the Spanish academic has been traditionally rooted.

In this study, we have provided evidence of how a set of coordinated actions, the Programme of Women’s Dialogic Action, when put into dialogue with diverse members of the public (women, men, public actors) can achieve unprecedented social and political impacts. The three core characteristics of ProWomenDialogue made all the actions carried out under it reach different social agents, from politicians to ethnic minorities, homemakers and union representatives, hence, opening up the debate and speaking up about a topic on which very few dared to speak before, but which within the academic realm everyone knew existed.

Many female researchers have left brilliant positions due to being victims of sexual harassment and not finding support during the process. Currently, thanks to the legislation passed (4/2007), most universities in Spain have Committees of Equality and Protocols in which procedures in these cases are established. Many cases have been in the media since the first report of sexual harassment by a catedrático, a fact not even imagined in Spanish academia two decades ago. Beyond this, survivors are actively engaged in prevention campaigns on different campuses and sharing their stories in national TV programs, radio, and social networks, explaining this reality at the university, and also showing the possibilities to report, face revictimization, and become successful in their careers. The #MeToo movement and the #TimesUp campaign are also examples of this empowerment. For the first time in Spanish academia, students and researchers are joining up with international and grassroots associations and networks. An example is the American End Rape on Campus (EROC) network, or the grassroots-based Roma Association of Women Drom Kotar Mestipen, committed to the creation of safer university contexts, which Roma families can trust, thus empowering Roma girls to attend and succeed. In this regard, due to the action of ProWomenDialogue, in Spain, there is the first junior female Romani researcher, SAPPHO member, and a referent for the Spanish Roma community, undertaking ground-breaking scientific contributions to both the Romani Studies and healthcare field [52].

Drawing on the SIOR criteria, ProWomenDialogue is connected to the UN Sustainable Development Goal 5: Achieve gender equality and empower all women and girls, undoubtedly making steps forward to close the gender gap in academia (criteria 1). The political and social impacts accomplished have improved the initial stage in Spanish academia (criteria 2), in which the situation was denying the very existence of sexual harassment and, when cases were known, they were hidden and treated as private issues of the Departments. Regarding replicability (criteria 3), Committees of Equality and Protocols now exist in most public universities and are being created in private ones. The actions of ProWomenDialogue have been widely published in different sets of sources, including scientific journals and governmental and non-governmental official bodies. Finally, transformations achieved are shown to be sustainable (criteria 5), and also have an impact on independent scientific institutions. For instance, ProWomenDialogue has promoted the modification of the Code of Ethics of Scientific Associations, including the topics of sexual harassment and isolating gender violence (e.g., Catalan Sociological Association, Multidisciplinary Association for Educational Research, and the European Sociological Association). In meeting all these criteria and revealing their impact, ProWomenDialogue can be considered a Successful Action in the struggle against sexual harassment in science and, therefore, transferable to other contexts.

## 5. Conclusions

Gender violence is still a problem, even after the implementation of many measures and legislation, and protections. The COVID-19 pandemic has made this situation even harder. Harassment interferes with the well-functioning of society, and with a person’s ability to learn and fulfill a proper life. High numbers show that, despite all the measures that are in place, cases are not decreasing, so the protection of direct survivors and those who support them is urgent [17]. There are many reasons why sexual assault victims choose not to report, including fear of reprisal, feeling it is a personal matter, not believing it is serious enough, shame, self-blame, and anxiety about not being believed [24]. The response to a survivor’s first disclosure may influence the survivor’s decision to get help or report to authorities. If the first reaction to their disclosure of sexual violence is doubt or disrespect, the survivor is less likely to seek help. Bystanders’ reactions are also influenced by potential reprisals or fear [53].

ProWomenDialogue has achieved an unprecedented transformation in both Spanish universities and science, breaking the silence on sexual harassment. Sexual harassment has been a barrier to the career promotion of many female researchers and has even led them to quit academia. It has been shown that beyond formal mechanisms, informal ones have played a key role. The MeToo University network, also inspired by the ProWomenDialogue, managed to inspire many survivors within academia. The political impact led to legislation that made compulsory the creation of equality committees and protocols against sexual harassment. Social impact, aligned with SDG 5, inspires the reduction of GBV. In addition, the Isolating Gender Violence legislation places Spain as a pioneer in legislating the need to protect the protectors to help encourage bystanders to become upstanders, which is key to contributing to overcoming GBV. Thus, ProWomenDialogue embodies a Successful Action against sexual harassment, presenting their features as recommendations to potentially be transferred to other contexts [54].

As shown, after the first relevant political impact of the programme (additional provision introduced in the Organic Law 4/2007 regarding ‘Units of Equality’), there were diverse policy and social actions about this issue, such that it is now constantly present in the media. Drawing from existing evidence on the importance of the joint actions carried out with egalitarian men within academia, future research needs to better explore the role of New Alternative Masculinities [55] in enhancing women’s scientific career success, how these types of masculinities develop their leadership, their position against harassment, and how they promote female colleagues, among others.

## Data Availability

The authors confirm that the data supporting the findings of this study are available within the article. For additional information requested in this sense, do not hesitate to contact the corresponding author.
